# A case report of multicentric reticulohistiocytosis with atypical cutaneous presentation

**DOI:** 10.3389/fimmu.2024.1344313

**Published:** 2024-02-14

**Authors:** Xiangru Chen, Lin An, Zhongmin Jiang, Yuxi Jia

**Affiliations:** Department of Dermatology, China-Japan Union Hospital of Jilin University, Changchun, Jilin, China

**Keywords:** multicentric reticulohistiocytosis, gouty arthritis, nodular xanthomatosis, immunohistochemistry, differential diagnosis

## Abstract

Multicentric reticulohistiocytosis (MRH) is a rare systemic disorder characterized by histiocytic hyperplasia that mainly involves the skin, mucous membranes, and joints. The typical clinical features include papules, nodules, and arthritis. MRH lesions are relatively extensive but small and scattered. Joint inflammation is characterized by diffuse symmetric polyarthritis as the first symptom, which can be severe and disabling due to destructive joint changes. MRH is easily misdiagnosed in clinical practice. Here, we report the case of an elderly male patient who presented with polyarticular pain in the hip and interphalangeal joints as the first manifestation, followed by the development of large, isolated, bulging skin nodules, which are atypical MRH lesions. This is rare in all MRH case reports, and we made the correct diagnosis by combining skin histopathology, immunohistochemistry, and other clinical examinations. We performed surgical treatment on the local skin lesions of this patient. This case suggests that clinicians should actively correlate the condition and accurately diagnose MRH when encountering atypical skin changes or other diseases as the first symptom and explore the mechanisms of MRH and other clinical manifestations.

## Introduction

MRH, also known as lipoid cutaneous arthritis, is a disease characterized by cutaneous and mucosal nodules with destructive arthritis. The first case that could be described as MRH was documented by Weber and Freudenthal in 1937 (1), and it was formally named “multicentric reticulohistiocytosis” by Goltz and Laymon in 1954 (2). An association between MRH and underlying malignancies and autoimmune diseases has been reported in 30% and 15% of cases, respectively (3). Its prevalence is two to four times higher in women than in men (4).

Here, we report a case of MRH with concomitant large, isolated, and elevated cutaneous nodules accompanied by hip and interphalangeal joint pain. The atypical clinical presentation of the patient’s skin and the low incidence of hip pain predisposed the patient to misdiagnosis, making the diagnosis of MRH challenging.

## Case description

The patient was a 70-year-old Chinese man who presented to our dermatology clinic in October 2023 with the main complaint of “a skin nodule that appeared in the right groin 1 year ago.” One year prior, a dark yellow-red, soybean-sized skin nodule had appeared in their right groin without any obvious triggers or conscious symptoms. The nodule gradually increased in size to that of a fava bean. Recently, the nodule had often rubbed against clothing, and the surface of the lesion was broken and scabbed, prompting the patient’s referral to our department. A review of their medical history revealed that the patient had red, swollen, and hot pain in the interphalangeal joints of both hands 2 years prior without any obvious triggers, as well as pain and skin swelling in the right hip joint 1 year prior, which was not accompanied by morning stiffness or activity limitation. They were diagnosed with “gouty arthritis” in the Department of Rheumatology and Immunology of the hospital. The patient was provided etoricoxib, febuxostat, and flurbiprofen cataplasms for symptomatic treatment, and their joint pain improved somewhat, but it was recurring and worsened gradually. During the course of the disease, the patient had no fever, fatigue, weight loss, or other symptoms, and they had no infectious diseases or family history.

**Figure 1:**
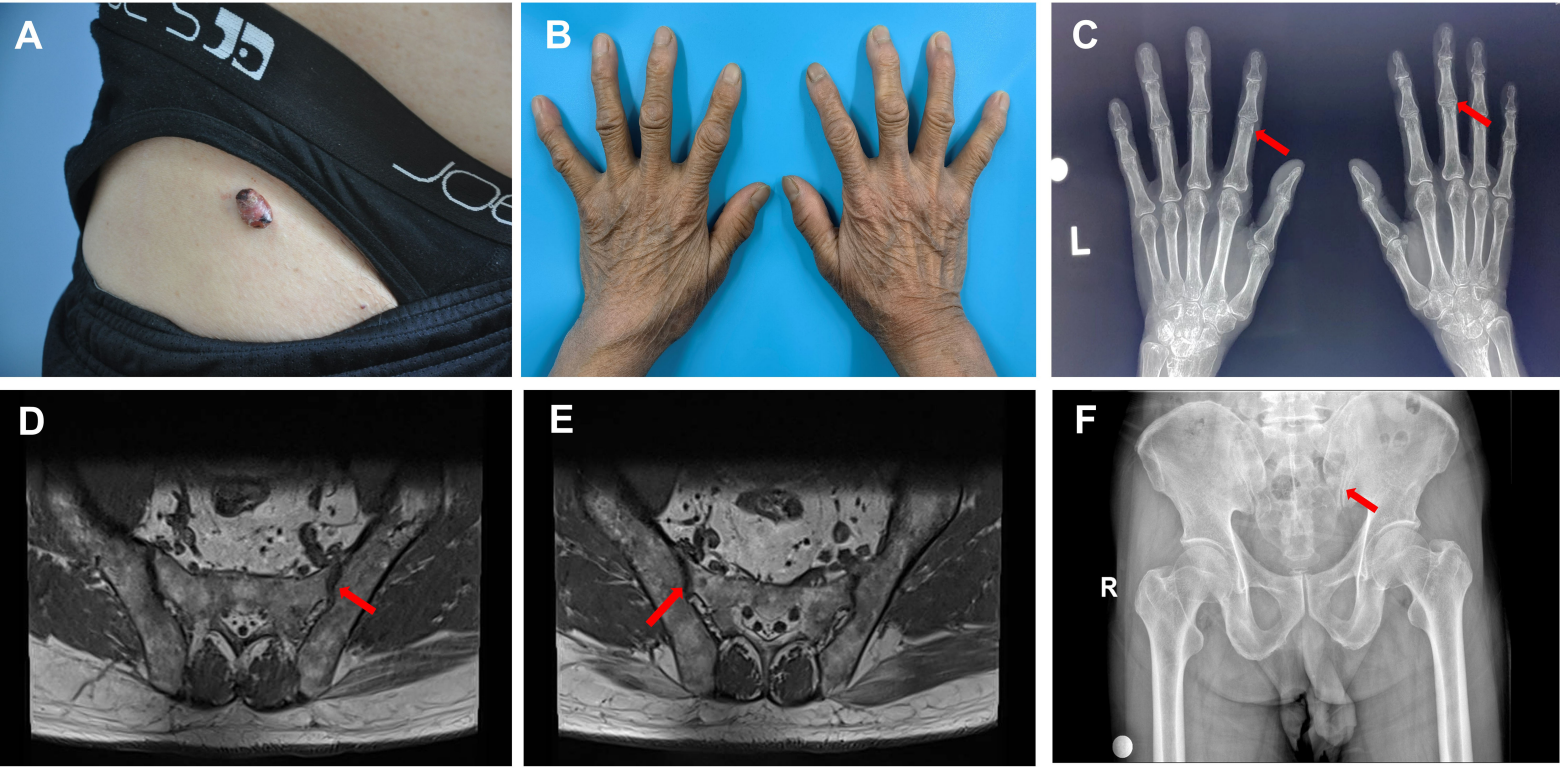
Skin changes and imaging in the patient with multicentric reticulohistiocytosis. **(A)** Skin lesions in the right inguinal region. **(B)** Mild swelling of the interphalangeal joints of both fingers of the patient, with no obvious deformation. **(C)** Mild bony changes at the edges of the interphalangeal joints indicated by the red arrow on the X-ray, and mild narrowing of the interphalangeal joint space. **(D)** MR scan of the sacroiliac joint, with the red arrow indicating lipid deposition in the hip joint. **(E)** MR scan of the sacroiliac joint, with the red arrow indicating lipid deposition in the hip joint. **(F)** Pelvic X-ray, with the red arrow indicating degenerative changes in the hip joint with decreased cystic density.

Physical examination indicated no palpable enlargement of the superficial lymph nodes throughout the body, and the muscle strength of the limbs was grade V. There was physiological curvature of the cervical, thoracic, and lumbar vertebrae, an absence of pressure or tenderness of the cervical and thoracic spinous processes, no limitation of cervical spine activity, and no reduction in lumbar spine anterior flexion, posterior extension, or lateral flexion activity. A dermatologic examination revealed a hemispherical, elevated, isolated nodule measuring 1.8 × 1.5 × 1.0 cm^3^ in the right groin, which was dark yellow-red in color and had a smooth surface, brown crusts in some areas, and clear borders. The texture was hard; it exhibited good mobility with no apparent adhesion with the underlying tissue, and there was no tenderness observed (**Figure 1A**). The distal interphalangeal joints of both hands were mildly swollen, with limited movement and tenderness (**Figure 1B**). No abnormalities were observed in any of the remaining skin areas.

We performed relevant laboratory and auxiliary tests, and we found that the patient had elevated levels of lipids and uric acid, total cholesterol (CHOL) of 6.62 mmol/L, low-density lipoprotein cholesterol (LDL-C) of 4.27 mmol/L, lipoprotein-a (LP-a) of 45.98 mg/dL, and uric acid in renal function tests of 465.58 μmol/L and an erythrocyte sedimentation rate of 16 mm/h. The test results of their blood and urine routine, C-reactive protein, antinuclear antibody series (anti-dsDNA, anti-U1-RNP, anti-Sm, anti-SSA, anti-SSB, anti-Scl-70, anti-RO-52, anti-Jo-1, anti-CENPB, anti-PCNA, anti-PM-Scl, anti-histone, anti-nucleosome, ANA screen, ANA karyotype, anti-ribosomal P protein, and anti-AMA-M2), anti-CCP antibody, HLA-B27 gene, tumor-related markers (AFP, CEA, CA199, CA724, CA125, PSA, pPSA, SCCA, ProGRP, Cyfra211, and CA242), rheumatoid factor (RA), immunoglobulin, complement, liver function, blood glucose, and ionic electrolytes were normal.

Orthopantomographic radiography of both hands revealed mild bony changes at the edges of the interphalangeal joints and narrowing of the interphalangeal joint space (**Figure 1C**). MR scan of the sacroiliac joints revealed degenerative changes in the bilateral sacroiliac joints and localized lipid deposits in the sacrum and bilateral iliac joints (**Figures 1D, E**). Pelvic orthopantomography revealed degenerative changes in the hip joints (**Figure 1F**). Chest computed tomography (CT) revealed fibrous striations in the inferior lingual segment of the upper lobe of the left lung, a dorsal pendular effect of the lower lobe of both lungs, and cholecystitis. Cranial magnetic resonance (MR) cerebral functional imaging (DWI) indicated subacute-stage lacunar cerebral infarction in the right basal ganglia region. A bone density examination revealed a lumbar spine T-score of 0.9 and right and left hip joint T-scores of −0.5 and −1.3, respectively (T-score reference value: >1 is normal; −1 to −2.5 reflects bone loss; and <−2.5 indicates osteoporosis). Cardiac ultrasonography revealed left atrial enlargement, left ventricular hypertrophy, aortic atherosclerosis, widening of the ascending aorta, and mild regurgitation of the mitral, tricuspid, aortic, and pulmonary valves. Double lower-extremity arterial and venous ultrasonography revealed double lower-extremity arteriosclerosis. Ultrasound of the liver, gallbladder, pancreas, spleen, kidney, and ureter revealed mud-like stones in the gallbladder; a cyst in the left kidney; and no obvious abnormalities in the liver, bile ducts, pancreas, spleen, right kidney, or bilateral ureters.

**Figure 2:**
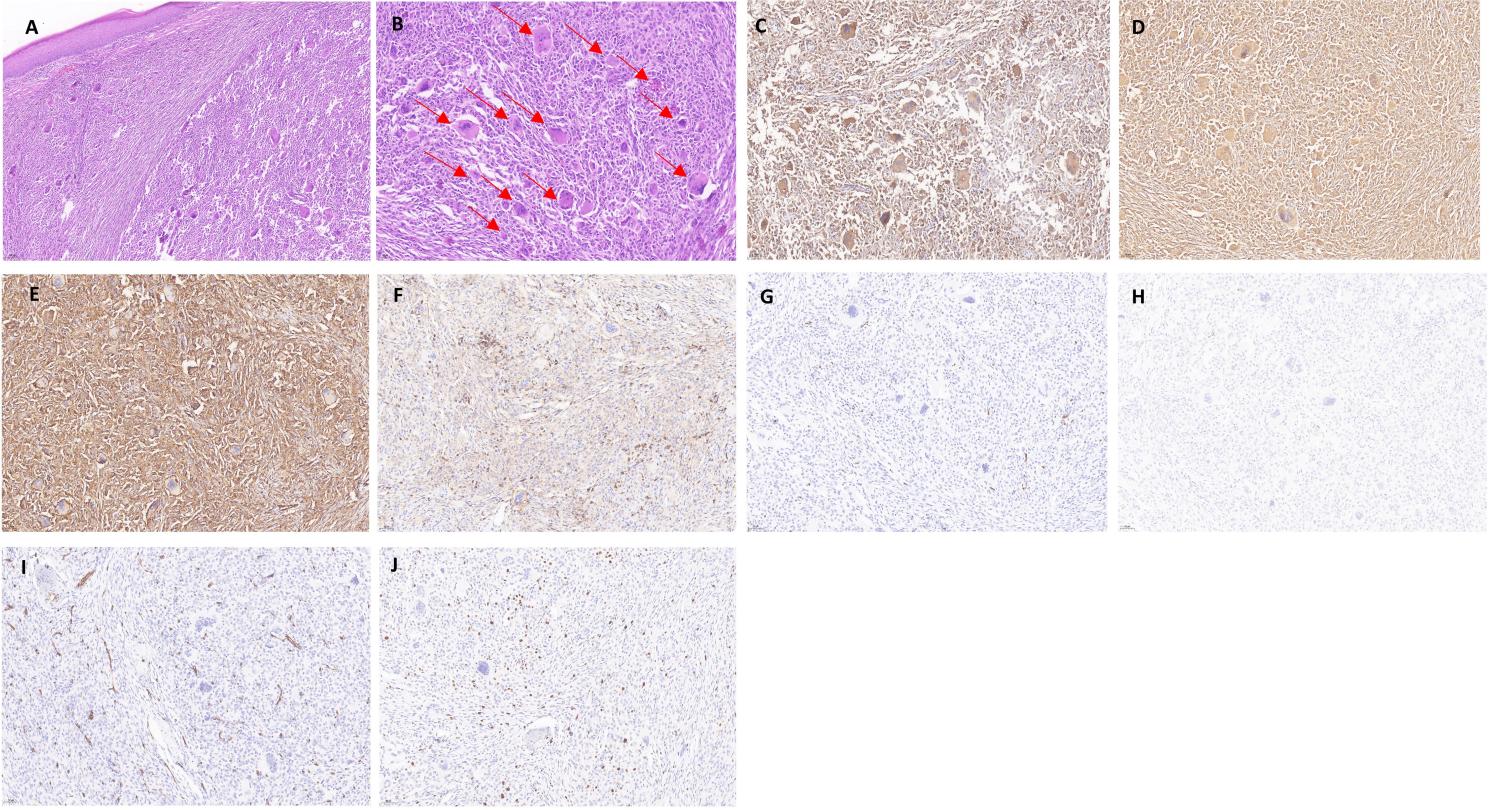
Histopathology and immunohistochemistry of the dermis of the patient with multicentric reticulohistiocytosis. **(A)** Mild atrophy of the epidermis, with a large number of histiocytes in the dermis, locally foamy (HE × 100). **(B)** Diffuse histiocyte infiltration in the dermis, accompanied by a large number of multinucleated giant cells in a “hairy glass”-like pattern, which is indicated with a red arrow (HE × 200). **(C–J)** Immunohistochemical staining for CD68 (diffuse +), vimentin (diffuse +), CD31 (diffuse +), and Vimentin (diffuse +). CD68 (diffuse +), vimentin (diffuse +), CD31 (diffuse +), LCA (CD45) (+), S-100 (−), CD1a (−), CD34 (−), and Ki-67 positivity of approximately 5%–10% (× 200).

By skin histopathology, diffuse histiocyte infiltration was seen in the dermis, with pink histiocytes, with a uniform morphology, and eosinophilic “hairy glass”-like multinucleated giant cells with clear borders (**Figures 2A, B**). Immunohistochemistry revealed CD68 (diffuse +), vimentin (diffuse +), CD31 (diffuse +), LCA (CD45) (+), S-100 (−), CD1a (−), CD34 (−), and Ki-67 positivity in approximately 5%–10% of the patients (**Figures 2C–J**).

**Figure 3:**
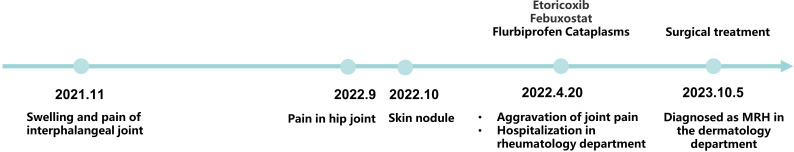
Schematic diagram of the clinical course of the patient.

Without knowledge of the patient’s history of polyarthralgia, it would have been difficult for us to make a definitive diagnosis based on the skin changes alone. Our follow-up of the patient comprised detailed histopathological, immunohistochemical, and other investigations, and the patient was ultimately diagnosed with MRH. The skin nodule in the right groin was surgically excised, and the patient was satisfied with the surgical outcome. In response to the patient’s polyarticular symptoms, we devised a systematic treatment program consisting of methotrexate combined with non-steroidal anti-inflammatory drugs. However, the patient refused the treatment for personal reasons and is now under close follow-up observation. The clinical course of the patient is shown in **Figure 3**.

## Discussion

The cutaneous manifestations of MRH are mainly reddish-yellow or reddish-gray papules and nodules that are scattered and hard, usually ranging from a few millimeters to several centimeters in diameter. They are primarily located on the face and dorsum of the hands but can also involve the arms, scalp, and neck. The papules near the proximal nail folds and the nostrils can undergo “coral bead”-like changes and, in severe cases, may exhibit “lion face” changes, which are the characteristic changes of MRH (5), and the nodules have a cephalad-caudal distribution, with a significantly reduced number in the lower part of the trunk and the lower limbs (6). In this case, the skin changes appeared in a rare site—the inguinal region—and the nodules were large and isolated, which is also rare in clinical practice.

MRH joint symptoms are obvious: more than 50% of cases have diffuse symmetrical polyarthritis as the first symptom, most often involving the hands and feet, including the distal interphalangeal joints, joint redness, swelling, pain, fluid accumulation in the early stage, and joint deformity or even disfigurement in the late stage; it can also involve the joints of the limbs, spine, and temporal and mandibular joints (7, 8). Markku et al. systematically reviewed 43 patients with MRH and inflammatory arthritis. Of the 43 patients with MRH, 42 (97%) had finger involvement, 22 (51%) had distal interphalangeal joint involvement, 22 (51%) had proximal interphalangeal joint involvement, 12 (25%) had metacarpophalangeal joint involvement, and 4 (9%) had carpometacarpal joint involvement. Other joint sites included the knee in 33 patients (77%), wrist in 21 (49%), shoulder in 19 (44%), elbow in 16 (37%), ankle in 10 (23%), hip in 6 (14%), foot in 5 (11.6%), and acromioclavicular joint in 2 (4.6%) (9).

In addition to the changes that occur in the skin and joints, MRH can also affect other organs such as the heart, lungs, urinary system, and thyroid gland; in severe cases, pericardial effusion, heart failure, and pulmonary fibrosis can occur (10, 11). MRH can indicate a potential risk for the development of neoplasms, and the most commonly reported neoplasms in the literature include breast, lung, liver, ovary, colon, pancreatic, and kidney cancers and other related neoplasia (12–15).

The diagnosis of MRH must be closely associated with clinical manifestations and histopathological changes. Histopathological examination of the skin reveals many large, malformed, multinucleated histiocytes with eosinophilic cytoplasm, fine granules in the form of “ground glass,” and nuclei that are rounded or oval in shape, randomly arranged in varying numbers (9). Immunohistochemical detection of infiltrating cells was positive for CD68, vimentin, CD31, and LCA (CD45) (16–18) and negative for the Langerhans histiocyte markers S-100, CD1a, and CD34 (19). Mohsin et al. have suggested that MRH may be caused by cytokine stimulation of histiocytes, resulting in increased nuclear schizophrenia and an increased K-index. Moreover, increased nuclear schizophrenic images and increased Ki-67 positivity may be indicators of the response to acute disease deterioration (20). Therefore, detection of Ki-67 is also necessary. In addition, dermoscopy can be helpful in diagnosing MRH. The appearance of yellow or orange plaques under dermoscopy is a high indication of dermal tissue cell infiltration. It can also be shown as yellow surface papules, cocoon structure, white unstructured areas, and linear skin dilatation (21, 22).

Notably, the patient in this case had lesions with morphologies similar to nodular xanthomatosis, as well as mildly elevated lipid levels and lipid deposition on MR of the hip, suggesting that MRH is closely related to lipid metabolism. Although previous studies have reported the presence of lipids in the cytoplasm of giant cells at the site of MRH lesions by histochemistry and electron microscopy, it has been demonstrated that lipid deposition does not play a major role in the pathogenesis of the disease and that intracellular lipid deposition in tissues appears to be a non-specific degenerative process rather than a manifestation of a lipid storage defect (23–25). This patient should be differentiated from patients with nodular xanthomatosis and gouty arthritis. This elderly man had a history of elevated uric acid levels without a high-purine diet, and their joint pain recurred and progressively worsened despite slight improvement after treatment for gout. Gout does not cause skin or other organ damage, except for joint changes, and the histology of the two diseases is completely different. Nodular xanthomatosis has no obvious destructive joint lesions, whereas characteristic histiocytes and multinucleated giant cells are rare. Because the lesion in this case was isolated, surgical excision was the most direct means for treatment; however, the patient’s symptoms of polyarthralgia continued to recur and gradually worsened, and they refused further treatment for personal reasons.

There is no clear treatment protocol for MRH, and its treatment is challenging. First-line treatments usually include methotrexate, glucocorticoids, or non-steroidal anti-inflammatory drugs. Second-line therapeutic options are mainly immunosuppressive, and the more widely used ones include TNF-α inhibitors. Switching to a different TNF-α inhibitor can be an effective alternative if the first-line medication is not effective (16, 26, 27). Recent reports indicate that upadacitinib, a JAK inhibitor, may be effective in treating MRH. Upadacitinib can reduce the level of IL-6 mediated by JAK-1 in patients with MRH, thereby slowing the degree of inflammation. It has also been suggested that MRH should be treated as a tumor disease, and molecular-targeted therapy is recommended when necessary (28). In this case, the patient declined further systemic treatment for personal reasons, and we will follow up with the patient.

MRH, although low in terms of its incidence, may cause severe arthropathy or entail a risk of other systemic tumors and thus require the accumulation of more samples along with empirical treatment and close follow-up of patients to provide a more rational treatment plan.

## Conclusion

The patient had a large raised nodule resembling nodular xanthomatosis, as well as pain in the hip and interphalangeal joints. These symptoms are inconsistent with the common clinical manifestations of MRH and must be clinically differentiated from lipid metabolism disorders and other types of arthritis. By reporting this case and reviewing the literature on MRH, this article aims to help clinicians improve their understanding of this rare disease. It suggests that when the initial diagnosis does not explain the full picture of the disease, further evidence is needed to confirm the final diagnosis.

## Data availability statement

The original contributions presented in the study are included in the article/**Supplementary Material**. Further inquiries can be directed to the corresponding author.

## Ethics statement

Written informed consent was obtained from the individual(s) for the publication of any potentially identifiable images or data included in this article.

## Author contributions

XC: Conceptualization, Investigation, Software, Writing – original draft. LA: Data curation, Methodology, Supervision, Writing – original draft. ZJ: Formal analysis, Project administration, Writing – review & editing. YJ: Resources, Visualization, Writing – review & editing.
