# Supplementary material for: A case report of multicentric reticulohistiocytosis with atypical cutaneous presentation
Source: Front Immunol. 2024 Feb 14;15:1344313. doi: 10.3389/fimmu.2024.1344313 (PMC10903083; doi:10.3389/fimmu.2024.1344313)
Supplement: Supplementary file 1 [file DataSheet_1.pdf]

CARE case report guidelines

CARE Checklist of information to include when writing a case report

(cc) BY-NC-ND

Topic	Item	Checklist item description	Reported on Line
Title	1	The diagnosis or intervention of primary focus followed by the words "case report"	Title
Key Words	2	2 to 5 key words that identify diagnoses or interventions in this case report, including "case report"	Key woeds
Abstract (no references)	3a	Introduction: What is unique about this case and what does it add to the scientific literature?	the seventh sentence of the abstract
	3b	Main symptoms and/or important clinical findings	the sixth sentence of the abstract
	3c	The main diagnoses, therapeutic interventions, and outcomes	the senventh sentence of the abstract
	3d	Conclusion—What is the main "take-away" lesson(s) from this case?	the ninth sentence of the abstract
Introduction	4	One or two paragraphs summarizing why this case is unique (**may include references**)	Introduction
Patient Information	5a	De-identified patient specific information.	the first sentence of the Case description
	5b	Primary concerns and symptoms of the patient.	the first paragraph of the Case description
	5c	Medical, family, and psycho-social history including relevant genetic information	the last sentence of the of the first paragraph in Case description
	5d	Relevant past interventions with outcomes	the seventh sentence of the first paragraph in Case description
Clinical Findings	6	Describe significant physical examination (PE) and important clinical findings.	the second paragraph of the Case description
Timeline	7	Historical and current information from this episode of care organized as a timeline	the last sentence of the Case description
Diagnostic Assessment	8a	Diagnostic testing (such as PE, laboratory testing, imaging, surveys).	the The third, fourth, and fifth paragraphs of the Case description
	8b	Diagnostic challenges (such as access to testing, financial, or cultural)	the last paragraph of the Case description
	8c	Diagnosis (including other diagnoses considered)	the last paragraph of the Case description
	8d	Prognosis (such as staging in oncology) where applicable	the last sentence of the Case description
Therapeutic Intervention	9a	Types of therapeutic intervention (such as pharmacologic, surgical, preventive, self-care)	the last paragraph of the Case description
	9b	Administration of therapeutic intervention (such as dosage, strength, duration)	the last paragraph of the Case description
	9c	Changes in therapeutic intervention (with rationale)	the fourth sentence of the last paragraph in Case description
Follow-up and Outcomes	10a	Clinician and patient-assessed outcomes (if available)	the fourth sentence of the last paragraph in Case description
	10b	Important follow-up diagnostic and other test results	NA
	10c	Intervention adherence and tolerability (How was this assessed?)	the last sentence of the Case description
	10d	Adverse and unanticipated events	NA
Discussion	11a	A scientific discussion of the strengths AND limitations associated with this case report	the fourth paragraph of the discussion
	11b	Discussion of the relevant medical literature **with references**.	The first, second, and third paragraphs of the discussion
	11c	The scientific rationale for any conclusions (including assessment of possible causes)	The first, second, third and fourth paragraphs of the discussion
	11d	The primary "take-away" lessons of this case report (without references) in a one paragraph conclusion	the last paragraph of the discussion
Patient Perspective	12	The patient should share their perspective in one to two paragraphs on the treatment(s) they received	the fourth sentence of the last paragraph in Case description
Informed Consent	13	Did the patient give informed consent? Please provide if requested	Yes ☑ No ☐
